# The fundamental theorem of natural selection with mutations

**DOI:** 10.1007/s00285-017-1190-x

**Published:** 2017-11-07

**Authors:** William F. Basener, John C. Sanford

**Affiliations:** 1 Rochester Institute of Technology, 1 Lomb Memorial Drive, Rochester, NY 14623 USA; 2 Horticulture Section, NYSAES, 630 West North Street, Geneva, New York 14456 USA

**Keywords:** Population genetics, Population dynamics, Mutations, Fitness, Fisher, Fundamental theorem of natural selection, Natural selection, Mutational meltdown, 92D15, 92D25, 92D10

## Abstract

The mutation–selection process is the most fundamental mechanism of evolution. In 1935, R. A. Fisher proved his fundamental theorem of natural selection, providing a model in which the rate of change of mean fitness is equal to the genetic variance of a species. Fisher did not include mutations in his model, but believed that mutations would provide a continual supply of variance resulting in perpetual increase in mean fitness, thus providing a foundation for neo-Darwinian theory. In this paper we re-examine Fisher’s Theorem, showing that because it disregards mutations, and because it is invalid beyond one instant in time, it has limited biological relevance. We build a differential equations model from Fisher’s first principles with mutations added, and prove a revised theorem showing the rate of change in mean fitness is equal to genetic variance plus a mutational effects term. We refer to our revised theorem as the fundamental theorem of natural selection with mutations. Our expanded theorem, and our associated analyses (analytic computation, numerical simulation, and visualization), provide a clearer understanding of the mutation–selection process, and allow application of biologically realistic parameters such as mutational effects. The expanded theorem has biological implications significantly different from what Fisher had envisioned.

## Introduction

R. A. Fisher was one of the greatest scientists of the 20th century. He is considered to be the singular founder of modern statistics and simultaneously the principle founder of population genetics (followed by Haldane and Wright). Fisher was the first to establish the conceptual link between natural selection and Mendelian genetics. This paved the way for what is now called neo-Darwinian theory.

At the heart of Fisher’s conception was his famous fundamental theorem of natural selection (Fisher’s Theorem). Fisher’s Theorem, published in his text *The Genetical Theory of Evolution* (Fisher 1930), showed that given a population with pre-existing genetic variants (i.e., Mendelian alleles) the population’s mean fitness will increase. Not only will mean fitness increase, the rate of increase will be proportional to the genetic variance for fitness within the population at any given time. This constitutes a proof that natural selection leads to increasing fitness in idealized Mendelian genetics, although it is often overlooked that Fisher’s theorem does not consider mutations and without newly arising variants natural selection can only lead to stasis.

By itself, Fisher’s Theorem seems obvious and of little significance. The impact of the theorem came from the following two points.

(A) Fisher conceptually linked natural selection with Mendelian genetics, which had not been done up to that time.
(B) Fisher assumed that, when combined with a constant inflow of new mutations, his theorem guaranteed unbounded increase of any population’s fitness. Therefore in his mind his theorem constituted a mathematical proof of Darwinian evolution.

At the time of Fisher’s work, there were two competing schools of thought about genetics and evolution (Plutynski 2006). The Biometric school viewed genetics as quantitative and continuous, fully understandable solely by statistical metrics and a vague notion of Darwinian gradualism. The Mendelian school of thought viewed inheritance as the transmission of discrete Mendelian units, hence evolution was thought to progress by discrete steps. In describing Fisher’s goal in his text, Plutynski writes, “His aim was to vindicate Darwinism and demonstrate its compatibility with Mendelism—indeed, its necessity given a Mendelian system of inheritance” (Plutynski 2006). Fisher wanted to show that the established reality of the discrete units of Mendelian inheritance did not undermine Darwinian evolution (as some were arguing), but actually supported it.

### Fisher’s derivation of how natural selection and Mendelian genetics can work together

Fisher’s model, and the assumptions he placed on his model system, have been investigated by numerous authors. It is generally accepted that while Fisher does not clearly state his assumptions about his system, it is possible to create a model system consistent with his work in which the proof of his theorem is valid. Price summarizes various perspectives on Fisher’s Theorem as (Price 1972):

Also, he [Fisher] spoke of the “rigour” of his derivation of the theorem and of “the ease of its interpretation”. But others have variously described his derivation as “recondite” (Crow and Kimura 1970), “very difficult” (Turner 1970), or “entirely obscure” (Kempthorne 1957). And no one has ever found any other way to derive the result that Fisher seems to state. Hence, many authors (not reviewed here) have maintained that the theorem holds only under very special conditions, while only a few (eg. Edwards 1967) have thought that Fisher may have been correct—if only we could understand what he meant!

It will be shown here that this latter view is correct. Fisher’s theorem does indeed hold with the generality that he claimed for it. The mystery and the controversy result from incomprehensibility rather than error.

Fisher’s model assumes many simplifying (but unrealistic) assumptions that define the limited generality that Price describes. For example, Fisher’s Theorem requires the assumption of zero dominance and zero epistasis. Price (1972) posits that Fisher defined dominance and epistasis to be environmental effects, which makes the theorem correct in this restricted level of generality, but limits its application as a fundamental rule affecting biological species as Fisher later claims. Ewens (1989) confirms the validity of Fisher’s Theorem in this level of generality. Also, Fisher’s definition of genetic variance uses a metric that changes with the population, thus his measure of genetic fitness is only applicable to a single moment in time, thwarting the development of a dynamic model of the evolution of the population (Price 1972; Ewens 1989). Fisher defines the expected value of fitness of an organism *y* to be

1.1 $$\begin{aligned} X(y) = \bar{m} + \sum _l Q_{l,a(y,l)} \end{aligned}$$

where $\bar{m}$ is the average fitness of the population, the sum is over every loci *l* in the genome, *a*(*y*, *l*) is the allele for the organism *y* at loci *l*, and $Q_{l,a}$ is the “increment” [Fisher’s terminology (Fisher 1930, p. 32)] associated with allele *a* at loci *l*, defined by Fisher to be the difference from the mean fitness that an organism will gain by having this allele at this locus. While Fisher does not provide a direct formula for the increments, Price (1972) suggests that they are the regression coefficients associated with the allele, defined by letting $P_{l,a}$ be the population of all organisms with allele *a* at locus *l*, $\# P_{l,a}$ be the number of organisms in population $P_{l,a}$, *m*(*y*) be the fitness of organism *y*, $\bar{m}$ be the mean fitness of the total population, and

1.2 $$\begin{aligned} Q_{l,a} = \sum _{y\in P_{l,a}} \frac{m(y)-\bar{m}}{\# P_{l,a}}. \end{aligned}$$

The genetic variance as defined by Fisher is the variance of the genetic fitness *X*(*y*) over all organisms *y* [See Price (1972) for a complete derivation from this perspective]. Because Fisher’s measure of genetic fitness of each organism *y* depends on the constituency of the population as a whole at that time, his theorem cannot be extended to a dynamic model over time. This, combined with his modeling ignoring important effects such as epistasis, does not invalidate Fisher’s theorem, but it makes his theorem inconsistent with his conclusion about how it applies as a universal law of evolution to all biological populations over time (Price 1972; Ewens 1989).

Fisher believed that his fundamental theorem applied to all species as a natural part of their function, “As each organism increases in fitness, so will its enemies and competitors increase in fitness”; (p. 41, Fisher 1930), not as providing a special case or just one piece of the change in fitness over time. Moreover, Fisher clearly claimed that his fundamental theorem should be as universal as entropy in thermodynamics (p. 36, Fisher 1930):

It will be noticed that the fundamental theorem proved above bears some remarkable resemblances to the second law of thermodynamics. Both are properties of populations, or aggregates, true irrespective of the nature of the units which compose them; both are statistical laws; each requires the constant increase of a measurable quantity, in the one case the entropy of a physical system and in the other the fitness, measured by m, of a biological population. As in the physical world we can conceive of theoretical systems in which dissipative forces are wholly absent, and in which the entropy consequently remains constant, so we can conceive, though we need not expect to find, biological populations in which the genetic variance is absolutely zero, and in which fitness does not increase. Professor Eddington has recently remarked that “The law that entropy always increases the second law of thermodynamics holds, I think, the supreme position among the laws of nature”. It is not a little instructive that so similar a law should hold the supreme position among the biological sciences.

Despite the limitations in Fisher’s theorem, Point (A) above (that natural selection can result in an optimization process of allele frequencies) is widely accepted. Thus, while his methods to compute fitness from the genetic level have not become universally accepted, his general conclusion concerning Point (A) has been accepted.

What is often overlooked is that without a constant supply of new mutations, selection can only increase fitness by reducing genetic variance (i.e., selecting away undesirable alleles, eventually reducing their frequencies to zero). This means that given enough time, selection must reduce genetic variance all the way to zero, apart from new mutations. According to Fisher’s Theorem, at this point effective selection must stop and fitness must become static. This evolutionary scenario only results in a minor increase in fitness followed by terminal stasis. Apart from a constant supply of new mutations, Fisher’s Theorem would actually suggest that “Mendelism has killed Darwinism” (Glick 2009, p. 265), a common view in Fisher’s time. This is precisely the opposite of what Fisher wanted to prove.

### Fisher’s theorem with mutations

In terms of Fisher’s primary thesis, we cannot overstate the essential role of new mutations and their fitness effects. Fisher’s theorem by itself actually shows that, apart from new mutations, a population can only optimize the frequencies of the pre-existing alleles, followed by stasis. Yet Fisher argued forcefully that his theorem was so fundamental in its nature, that it essentially guaranteed that any population would increase in fitness without limit (essentially constituting a mathematical proof that Darwinian evolution is inevitable). How could he make this argument? To make his theorem meaningful Fisher had to assume a constant supply of new mutations. He understood that both deleterious and beneficial mutations occur, but argued against the effects of deleterious mutations (p. 41, Fisher 1930):

If therefore an organism be really in any high degree adapted to the place it fills in its environment, this adaptation will be constantly menaced by any undirected agencies liable to cause changes to either party in the adaptation. The case of large mutations to the organism may first be considered, since their consequences in this connexion (sic) are of an extremely simple character. A considerable number of such mutations have now been observed, and these are, I believe, without exception, either definitely pathological (most often lethal) in their effects, or with high probability to be regarded as deleterious in the wild state. This is merely what would be expected on the view, which was regarded as obvious by the older naturalists, and I believe by all who have studied wild animals, that organisms in general are, in fact, marvellously (sic) and intricately adapted, both in their internal mechanisms, and in their relations to external nature. Such large mutations occurring in the natural state would be unfavourable (sic) to survival, and as soon as the numbers affected attain a certain small proportion in the whole population, an equilibrium must be established in which the rate of elimination is equal to the rate of mutation. To put the matter in another way we may say that each mutation of this kind is allowed to contribute exactly as much to the genetic variance of fitness in the species as will provide a rate of improvement equivalent to the rate of deterioration caused by the continual occurrence of the mutation.

He reasoned that mutations that were seriously deleterious would easily be selected away, and so could be ignored. Beyond this, he loosely suggested that the downward impact on fitness must be balanced by the upward impact on genetic variance. In mutation–selection population models, as described in Sect. 2, there is a balance between the downward effects of deleterious mutations and upward effect of selection that balances out in infinite population models but not in finite population models. Our main theorem, Theorem 2, provides the rate of change of mean fitness into two terms, the first being the genetic variance and the second being a decrease in fitness from mutations, and the two are not equal.

After arguing that large mutations are generally deleterious and can be ignored because they are self-eliminating, Fisher argues that mutations with small net effects have a nearly equal chance of being deleterious as being beneficial (p. 46, Fisher 1930):

Adaptation, in the sense of conformity in many particulars between two complex entities, may be shown, by making use of the geometrical properties of space of many dimensions, to imply a statistical situation in which the probability, of a change of given magnitude effecting an improvement, decreases from its limiting value of one half, as the magnitude of the change is increased. The intensity of adaptation is inversely proportional to a standard magnitude of change for which this probability is constant. Thus the larger the change, or the more intense the adaptation, the smaller will be the chance of improvement.

Having argued that the effects of large mutations can be ignored, he argues that the small mutations have a net effect that was effectively neutral (the net effect approaches zero as the size of the effect approaches zero), with $50\%$ of these mutations being beneficial and $50\%$ of being deleterious. Fisher does not consider any mutations other than those with large deleterious effects and those with small nearly neutral effects.

It is now clear Fisher was wrong regarding the effects of mutations. Research since that time, described in Sect. 2, shows that the mutations with intermediate fitness effects have the greatest impact on long-term fitness. This has been shown in models, demonstrated in laboratory experiments, and has led to antiviral therapies. At the time Fisher wrote, the distribution of mutational effects was not understood, and so his fundamental assumption was incorrect.

Fisher’s primary error was that he sincerely believed that mutations by themselves could continuously restore genetic variance without affecting fitness (and then selection could always translate the replenished genetic variance into increased fitness). It is very significant that new mutations were not part of Fisher’s mathematical formulation, he only added mutations as an informal corollary to his Theorem. Although Fisher did not explicitly make the distinction, for clarity we need to separate Fisher’s Theorem (no mutations included) from “Fisher’s corollary” (mutations included).

#### Fisher’s Corollary 1

Fisher’s fundamental theorem, plus a steady supply of new mutations, necessarily results in unbounded fitness increase, as mutations continuously replenish variance, and as selection continuously turns that variance into increased fitness.

The term “corollary” is justified here because Fisher believed that if Fisher’s fundamental theorem is true, then the corollary is true as a necessary logical consequence. Fisher never derived his corollary mathematically. Moreover, most modern evaluations of Fisher’s theorem focus on the theorem itself and do not address the role of mutations.

It has been observed that systems with more than one loci and recombination can have limit cycles and mean fitness (measured as mean reproduction rate) that is not strictly increasing (Karlin and Carmelli 1975; Hastings 1981), and periodic oscillations can occur in diploid models (Hofbauer 1985; Burger 1989). Following the approach of Price, the component of change in mean fitness due to natural selection is still equal to variance in genetic fitness but recombination and other factors can act as external variables, and so these special cases may not violate Fisher’s fundamental theorem of natural selection in its limited generality. However, these examples do violate how Fisher perceived universal applicability of the theorem in the sense of always increasing mean fitness. In this paper we address the mutation–selection process in the restricted setting that Fisher considered.

This paper show’s mathematically how Fisher’s Corollary depends upon the assumption that the net effect of new mutations must be effectively fitness-neutral. Even if Fisher had understood the nature of mutations, and had developed a mathematical model for the actual effect of mutations on fitness, there seems to be no clear way for him to incorporate that model into his original theorem. This is because his theorem is formulated to only consider modifications in frequencies of pre-existing alleles.

In order to understand Fisher’s theorem in light of newly arising mutations, we need to reformulate the original theorem to allow for incoming new mutations. Instead of building the model up from the genetic allele level, we consider the resulting fitness to be equal to the Malthusian growth rate of the population in its environment, such that a “*special example*” (Crow and Kimura 1970, p. 10) of Fisher’s theorem can be proven. This new version of the theorem includes an objective metric of fitness which allows for dynamic modeling of the mutation–selection process over time. In this special case, we are exchanging Fisher’s derivation of the theorem based upon pre-existing Mendelian alleles for a new derivation that has the ability to quantify fitness with an objective metric that can be applied to a changing population. The statement of Fisher’s fundamental theorem becomes “*the rate of change of fitness at any instant, measured in Malthusian parameters, is equal to the variance in fitness at that time*” (Crow and Kimura 1970, p.10).

The goal of this paper is to develop a version of Fisher’s theorem analogous to that presented by Crow and Kimura (1970), but with the additional capability of tracking the effects of mutations to new genetic varieties over time. This new formulation is proven as Theorem 2, where we derive a formula that gives the rate of change of mean fitness as a function of both the variance in fitness and the mutation effects on population fitness. In this manner, we provide the ability to mathematically analyze Fisher’s Corollary (Point (B)).

Since the premise underlying Fisher’s Corollary is now demonstrably wrong, it is a forgone conclusion that Fisher’s Corollary is false. Mutations are not effectively fitness-neutral, not even when all large deleterious mutations are eliminated by selection, so Fisher’s conclusion that natural selection with mutations *necessarily* results in increasing fitness is not true. In reality, the direction and rate of fitness change is the sum of two terms. One term is the upward effect of selection, which is proportional to genetic variance in fitness (as in Fisher’s original formulation). The other term is the net downward effect of mutations, which will depend on the exact distribution of mutational fitness effects and other biological factors affecting selection effectiveness. Our Theorem 2, which we call the fundamental theorem of natural selection with mutations, expands upon Fisher’s fundamental theorem of natural selection by incorporating into it the modern understanding of mutations.

## Mutation–selection models—a review of the literature

Since the early days of Fisher, Haldane, and Wright, a number of newer models for the mutation-selection process have been formulated. On a practical level it does not seem that most population biologists have actually believed that fitness could increase universally, continuously, and without bound. Indeed, the literature has reported many empirical and theoretical studies that indicate that fitness increase can be very problematic, and that fitness decline is a very real possibility for any population. It seems most population biologists have viewed Fisher’s theorem as being simply out of date and of modest historical interest. Yet theorems should not just fade away—mathematically they should be upheld, refuted, or corrected. Our goal is to correct and re-apply Fisher’s Theorem, such that it is consistent with real biology.

Most of the more recent mutation–selection models have a general framework which employs a method for describing all possible genotypes (called the state space), wherein organisms reproduce at rates proportional to the fitness determined by the parent genotype(s), and resulting progeny can be of a different genotype than the parents caused by mutations. Each model has its own set of variables that are studied and its own set of rules governing change over time.

Every model is only an approximation of some isolated subset of reality, and each model is only useful insofar as it: (1) includes the variables and rules to be studied and: (2) the rules governing change in the model accurately approximate the most important factors affecting change in reality. Simple rules make a model more mathematically tractable, but at the cost of utility as a useful model of reality. The general goal is to have rules that are as simple as possible, and yet capture all the driving factors contributing to the phenomena to be studied. In using a model to make general statements about behavior in reality, it is essential to consider the built-in assumptions implicit in the structure of the rules in a model.

### Deterministic versus non-deterministic models

Deterministic models are models in which all future behavior is determined by the current state of the system. In non-deterministic models (sometimes called agent-based methods), mutations and Mendelian genetic principles (which are non-deterministic on an individual organism level) are used to determine genetics of offspring from parents. Numerical simulations can simulate change in genetics over time, and there is almost no limit to the complexity of the simulation. As with all numerical simulations, general principles are not derived mathematically as much as are made as general observations from repeated experiments. Non-deterministic agent-based methods are at the extreme end of enabling the most accurate approximation of factors driving genetic change in real biological systems at the cost of little accessibly to proving mathematical principles or laws. Non-deterministic models can be used to explain what is likely (or unlikely) to happen given some underlying set of governing rules, but are unlikely to provide must-happen rules in the form of physical laws that Fisher sought in his fundamental theorem.

The ideal situation in using models to understand reality is to have phenomena that is observable in reality, observable in non-deterministic models, and that has underlying principles provable in deterministic models.

### Infinite population models

In this section we discuss models for mutation–selection that come under the general heading of infinite population models. In these models, the population size is held at carrying capacity but the population can be divided into infinitely small subsets. These models also have an explicitly defined state space describing the possible genotypes and mutations occuring but only between genes that are already present. Selection occurs as genetic varieties reproduce and compete at different rates depending on fitness, providing a first-principles approach to selection. These models are important for understanding the selection–mutation process as a population adapts to its environment, but do not provide direct insight into how a population forms new genotypes that may be more fit or less fit than the original population; no genes are lost and no new genes are created.

There are two main explicit models for the state space of genotypes upon which selection–mutation acts in infinite population models: measuring the frequency of alleles present and segregating population into subpopulations, each of which corresponds to a different genotype. Modeling from the allele frequencies is considered a population genetics approach because the focus is on the genetics and is the approach that Fisher used. Modeling each individual subpopulation by genotype is called the quasispecies theory because the population of a species is modeled as a cloud of separate genotypes each of which can mutate to any of the others, and is generally attributed to the work of Eigen in (1971), with the term quasispecies first used in Eigen and Schuster (1977). While the two approaches begin with different foundations, they are more appropriately described as two sides of the same coin as opposed to being entirely different models.

While these two types of models use differing descriptions of the state space, they both use equivalent rules for change over time: organisms reproduce proportional to fitness with mutations. Before introducing equations for specific models from each approach (which vary according to additional assumptions made), we discuss the context for using either approach.

The purpose of the allele-frequency approach (and other models based on the genetic components) is to investigate the change in the underlying genetics over time, as described in Burger (1989):

“Traditionally, models with only two alleles per locus have been treated. At the end of the fifties the first general results for multi-allele models with selection but without mutation were proved. In particular, conditions for the existence of a unique and stable interior equilibrium were derived and Fisher’s fundamental theorem of natural selection was proved to be valid (e.g. Mulholland and Smith 1959; Scheuer and Mandel 1959; Kingman 1961). It tells that in a one-locus multi-allele diploid model mean fitness always increases. It is well known now that in models with two loci or more this is wrong in general”.

An excellent exposition of the population genetics approach is provided in Burger (1989), which includes criteria for cases where a Lyapunov function [a function on the state space that is increasing with respect to time, also called a maximization principle (Hofbauer 1985)] may or may not exist. It is standard results that any system with a Lyapunov function (which may or may not be mean fitness) and a compact state space will have all solutions forward asymptotic to equilibria (Hofbauer 1985). In cases where the population approaches an equilibrium, new mutations tend to decrease fitness but are balanced by the selection process, called the *mutation–selection balance*. The mutation–selection balance is an important concept for real populations even though there is no guarantee of achieving such an equilibrium.

The objective in the quasispecies theory was originally to investigate error-prone self-replication of biological macromolecules with a focus on the origin on life. This theory has been applied with success to RNA viruses which replicate at high mutation rates and have extremely polymorphic populations (Pariente et al. 2001; Crotty et al. 2001; Grande-Perez et al. 2002; Anderson et al. 2004). The quasispecies approach enables investigation of the distribution of a population across a fitness landscape. Of primary importance is the measurement of when selection acting within a population will adapt effectively around the higher fitness varieties in the landscape vs. when mutations will cause the population to spread out across the landscape.

For a given fitness landscape, the mutation rate separating adaptation from spreading over low-fitness genotypes is called the *error threshold*. While the Quasispecies Equation always has an equilibrium and the form of the equation holds the total population fixed at carrying capacity, the idea that a high mutation rate causes the population to spread out over lower fitness genotypes has led to effective antiviral therapies in which the increase in mutation rate causes extinction of the population (Pariente et al. 2001; Crotty et al. 2001; Grande-Perez et al. 2002; Anderson et al. 2004). The infinite population models a priori prevent extinction (because of approximating assumptions in the models not present in the biological populations), and models that can exhibit such an extinction due to mutations will be provided in Sect. 2.3.

Although these two approaches use different models for the state space, they are more complementary than at opposition to each other. Because they both model change over time for organisms reproducing proportional to fitness with mutations, the behavior observed in each should at least be compatible. In Wilke (2005), the author explains “I review the pertinent literature, and demonstrate for a number of cases that the quasispecies concept is equivalent to the concept of mutation–selection balance developed in population genetics, and that there is no disagreement between the population genetics of haploid, asexually-replicating organisms and quasispecies theory”. The main difference between the approaches is how the measure that behavior and its effect on underlying variables (specifics of genetic variation in the population in the one case, and the distribution of the population over fitness landscape in the other). The population genetics approach allows the study of the effects of mutation–selection on allele frequencies and the quasispecies enables the study of the effects on the distribution of a population across a fitness landscape.

We now present equations for deterministic models for the mutation–selection process that assume an infinite population. The mutation process in the model is explicitly incorporated by a matrix of values that provide the mutation rate from one genotype (or allele) to a different one. As such, only mutations between pre-existing genotypes (alleles) are considered. Selection occurs via different reproduction rates for the genotypes (alleles) with the total population help at carrying capacity.

The infinite population assumption is implicit in the model; it is present by modelling each genotype frequency (or allele frequency) by a real number between 0 and 1. This makes the population “infinite” because a subset of the population corresponding to a genotype (or allele) can be a nonzero arbitrarily small fraction of the total population. Also implicit in the equations, connected to the infinite population assumption, is that every subpopulation is nonzero for all time making any form of extinction a priori impossible. In addition, these models only permit mutations among genetic varieties that are already present, limiting their utility for modeling ongoing change in a genes, either creating ongoing improvements as Fisher envisioned or building up deleterious mutations in fitness decline.

We start with the single locus case with multiple alleles $\{A_1,\ldots ,A_n\}$. Denote the frequency of allele $A_i$ by $p_i$ and $u_{ij}$ the mutation rate for allele $A_j$ to $A_i$. Denote the fitness of an organism with allele $A_i$ by $m_i$, and then the mutation–selection model from Crow and Kimura (1970) is

2.1 $$\begin{aligned} \frac{\text {d}P_i}{\text {d}t} = P_i(m_i-\bar{m}) + \sum _j u_{ij} P_j - P_i, \end{aligned}$$

where $\bar{m}=\sum _i m_i P_i$. In the terminology of Burger (1989), this is the “classical haploid one-locus multi-allele model with mutation and selection”. It is explicitly solvable with a unique forward-time stable equilibrium solution (Burger 1989), which is the mutation–selection balance.

For the one-locus diploid model with alleles $\{A_i,\ldots ,A_n\}$ and associated frequencies $\{p_1,\ldots ,p_n\}$, we define the fitness of an organism with allele $A_iA_j$ to be $m_{ij}=m_{ji}$. The marginal fitness of allele $A_i$ is then $m_i=\sum _{j=1}^n m_{ij}p_j$. The mean fitness is $\bar{m}=\sum _{i,j=1}^n m_{ij}p_ip_j$. We define $u_{ij}$ to be the mutation rate for allele $A_j$ to $A_i$ for $i\ne j$ satisfying $u_{ij}\ge 0$ and $\sum _{j=1}^n u_{ij} =1$ for all *j*. Then the one locus diploid model given in Crow and Kimura (1970) is:

2.2 $$\begin{aligned} \frac{\text {d}P_i}{\text {d}t} = P_i(m_i-\bar{m}) + \sum _j (u_{ij} P_j - u_{ji} P_i). \end{aligned}$$

The dynamics for the diploid model are more complicated (Burger 1989), and can include stable periodic orbits (Hofbauer 1985).

To describe the quasispecies model, we want to define the state space in terms of a (finite) set of different genetic sequences (genotypes). Suppose now that $P_i$ is the concentration of the $i\mathrm{th}$ genetic sequence, $m_i$ is the associated fitness, and that $Q_{ij}$ is the mutation probability from *j* to *i*. Then the quasispecies model as presented in Wilke (2005) is

2.3 $$\begin{aligned} \frac{\text {d}P_i}{\text {d}t} = P_i(m_i Q_{ii}-\bar{m}) + \sum _{j\ne i} m_j Q_{ij} P_j. \end{aligned}$$

From this form of the equation it is not difficult to see that this is equivalent to Eq. 2.1, with different interpretation of the constants. A more concise form of the quasispecies equation can be obtained by letting

2.4 $$\begin{aligned} \frac{\text {d}P_i}{\text {d}t} = \sum _j m_j Q_{ij} P_j - \bar{m}P_i. \end{aligned}$$

This is the form used in Nowak (2006), Chapter 3. This form has the advantage that all the reproduction–mutation creating genotype $P_i$ is in the first term, $\sum _j m_j Q_{ij} P_j$, and the second term ${-}\,\bar{m} P_i$ can be seen as the *normalization* term that keeps the total population constant. As with the haploid model 2.1, the quasispecies model has a unique for stable equilibrium solution.

The quasispecies equation allows examination of the distribution of the population across the fitness landscape. The distribution can be dense around the most fit genotypes (strongly adapted), or it can be spread out to include significant lower fitness genotypes (no adaptation). Selection tends to push the distribution to the higher fitness while mutations work in the opposite direction distribution the population evenly, the error threshold is the highest mutation rate at which adaptation occurs. Adaptation is only possible if the mutation rate per base is less than the inverse of the genome length using appropriate units (Nowak 2006).

The basic idea of Fisher’s Theorem, that mean fitness of a population is always increasing, is valid for the haploid one-locus and quasispecies models, but not for the diploid model. The basic idea of Fisher’s Corollary, that mutations add ongoing new varieties resulting in unbounded growth is untrue in all cases as they are asymptotic to a limit set.

More generally, given any model in this class of infinite population models in which mean fitness is always increasing, the mean fitness acts as a Lyapunov and all solutions are forward asymptotic to an equilibria (See Burger (1989) for specific criteria). Moreover, for any continuous mutation–selection model with non-decreasing continuous mean fitness function and a compact state space, there is a maximum possible mean fitness and all solutions will be asymptotic to a limit set [The state space will be compact if for example there is a finite upper bound on genome length. See Basener (2013b)]. In many cases, the existence of an increasing mean fitness mathematically precludes unbounded increase in mean fitness anticipated in Fisher’s Corollary.

The model we provide in Sect. 3 is not restricted in the genetic varieties that can be created via mutations, and thus can exhibit either ongoing increase or ongoing decrease in fitness depending on mutational effects. An error threshold type boundary between increasing and decreasing fitness is provided in Theorem 2.

### Finite population models

Finite population mutation–selection models are models with rules assuming a finite number of organisms. Since there is a limited number of genetic varieties to select among, selection becomes less effective. As a result, finite population models are able to produce realistic phenomena that is a priori impossible in infinite population models, most importantly the build-up of deleterious mutations over time.

An early significant step in finite population modeling was the simple thought experiment that in a small asexually reproducing population, no parent can have offspring more fit than the parent (beneficial mutations are insignificant compared to deleterious ones, and back mutations are rare). It is possible that through random chance the most fit class of organisms might not produce offspring as fit as the parents, and the genetic makeup of this class would be lost. The next most fit class could suffer the same fate, and so on until the population loses fitness needed for population survival. This was pointed out by Muller (1964), and termed “Mullerś Ratchet” by Felsenstein (1974), with each click of the ratchet being a loss of a most fit class.

The predominance of deleterious mutations over beneficial ones is well established. James Crow in (1997) stated, “Since most mutations, if they have any effect at all, are harmful, the overall impact of the mutation process must be deleterious”. Keightley and Lynch (2003) given an excellent overview of mutation accumulation experiments and conclude that “...the vast majority of mutations are deleterious. This is one of the most well-established principles of evolutionary genetics, supported by both molecular and quantitative-genetic data. This provides an explanation for many key genetic properties of natural and laboratory populations”. In (1995), Lande concluded that $90\%$ of new mutations are deleterious and, the rest are “quasineutral” (Also see Franklin and Frankham (1998)). Gerrish and Lenski estimate the ratio of deleterious to beneficial mutations at a million to one (Gerrish and Lenski 1998b), while other estimates indicate that the number of beneficial mutations is too low to be measured statistically (Ohta 1977; Kimura 1979; Elena et al. 1998; Gerrish and Lenski 1998a). Studies across different species estimate that apart from selection, the decrease in fitness from mutations is 0.2–2$\%$ per generation, with human fitness decline estimated at $1\%$ (See Lynch 2016; Lynch et al. 1999). Estimates suggest that the average human newborn has approximately 100 *de novo* mutations (Lynch 2016). Research using finite population models has been driven by the need to understand the impact of the buildup of deleterious mutations (called mutational load) in small populations of endangered species (See Lande 1995; Franklin and Frankham 1998). Of special interest is the mutational load in the human species given the relaxed selection due to social and medical advances (Kondrashov 1995; Crow 1997; Lynch 2016).

Lynch and Gabriel (1990), proposed a discrete-time (non-overlapping generations) model assuming that the effect of every deleterious mutation is equal to a value *s*, called the mutational effect or selection coefficient. The cumulative effects of mutations is assumed to be multiplicative, and so the fitness of a an organisms with *n* mutations is $W=(1-s)^n$ (assuming an initial well-adapted baseline fitness of 1). Reproduction proceeds assuming a carrying capacity of *K*; after reproduction if there are more than *K* individuals then only the most fit *K* are kept. In the model it is assumed that the initial population is adapted to its environment with little mutation variance, and is at carrying capacity, for example a newly arisen from an ancestral sexual species. Analysis of the model includes both numerical simulations and analytic computations.

Lynch and Gabriel show that the resulting dynamics has three quantitative phases. First, the mutations are rare and selection is effective, and the mutation number grows slowly to approach the expected drift–selection–mutation equilibrium value. During the second phase, mutations continue to accumulate at a nearly constant rate until the mean viability is reduced to 1 / *R* (less than one surviving progeny per adult, or equivalently a negative growth rate). From this point on, the population decreases in size which accelerates the decrease in fitness, resulting in “mutational meltdown”.

The Lynch–Gabriel mutation accumulation model enables estimation of important relationships—for example the time-to-extinction for varying values of the mutational effect *s* and the carrying capacity *K*, (Lynch et al. 1993, 1995a, b), and the time to extinction is approximately equal to the log of the carrying capacity Lynch et al. (1993). The models shows that “an intermediate selection coefficient (*s*) minimizes time to extinction” (Lynch et al. 1993). Mutations with an effect in this intermediate range are small enough to not be selected out but large enough to still impact overall fitness. The model was extended to similar conclusions for sexually reproducing organisms (Lynch et al. 1995b), with a longer time to extinction. It has been used as an important guide in managing endangered species (Franklin and Frankham 1998).

While the Lynch–Gabriel mutational accumulation model assumes all mutations have the same impact on fitness, in reality the possible effects of deleterious mutations is a distribution ranging from effectively neutral to lethal (Lynch et al. 1999):

A lethal mutation is one that in the homozygous state reduces individual fitness to zero, whereas deleterious mutations have milder effects and neutral mutations have no effect on fitness. Because the distribution of mutational effects is continuous, it is difficult to objectively subdivide the class of deleterious mutations any further.

Of critical importance are deleterious mutations that are small enough in effect to accumulate, which Kondrashov calls “very slightly deleterious mutations” (VSDMs) (Kondrashov 1995). He states, “The study of VSDMs constitutes one of the pillars of population genetics” and attempts to quantify the most dangerous range of VSDMs as follows: “deleterious mutations with an effect less than 1 / *G* (where *G* is the length of the genome) have little effect no fitness even in large numbers, and that deleterious mutations with an effect greater than $1/4N_e$ (where $N_e$ is the effective population size) will be eliminated via selection”. He then observes, “In many vertebrates $N_e\approx 10^4$, while $G\approx 10^9$, so this dangerous range includes more than four orders of magnitude” (Kondrashov 1995). Other authors (e.g. Butcher 1995) have described this dangerous range in terms of Mullerś ratchet; deleterious mutations with a larger effect give a larger turn of the ratchet at each click but have a slower rate of clicks (because they are more susceptible to selection), while mutations with smaller effects give a smaller rotation at each click but have a higher click-rate. The mutations with the greatest long term impact on fitness are in the middle range with the greatest net rotation rate of the ratchet. These are the mutations, like those in the range of values observed by Lynch et al that minimize time to extinction (Lynch et al. 1993), which can accumulate over time and have significant net impact over time on fitness. In (1997), Crow describes the effect of these mutations as follows:

...diverse experiments in various species, especially Drosophila, show that the typical mutation is very mild. It usually has no overt effect, but shows up as a small decrease in viability or fertility, usually detected only statistically. ... that the effect may be minor does not mean that it is unimportant. A dominant mutation producing a very large effect, perhaps lethal, affects only a small number of individuals before it is eliminated from the population by death or failure to reproduce. If it has a mild effect, it persists longer and affects a correspondingly greater number. So, because they are more numerous, mild mutations in the long run can have as great an effect on fitness as drastic ones.

Computations based on this dangerous range of mutation effects suggest that populations in general should be accumulating very slightly deleterious mutations sufficient to affect mean fitness. Kondrashov conjectured in (1995) that mutational epistasis and soft selection may stop the accumulation of VSDMs. Mutational epistasis is the concept that multiple mutations will have a larger cumulative effect then the sum (or product, depending on the model) of the individual effects. If mutations all have the same mutational effect *s* and it is in the dangerous range, then as mutations accumulate epistasis implies that the effects of additional mutations will have increasingly large effects, eventually so large as to be no longer in the dangerous region, able to be selected out of the population.

The mutation–accumulation model of Lynch et al. (Butcher et al. 1993; Lynch et al. 1995a, b) was extended from considering mutations with a single fixed effect *s* to a continuous range of possible effects by Butcher (1995). Butcherś model is exactly the Gabriel-Lynch model with two modifications. First, instead of all mutations having the same effect, a probability distribution for the possible mutational effects is used. Second, Butcher has an epistasis term in his fitness to account for the mutational epistasis. Butcher shows “epistasis will not halt the ratchet provided that rather than a single deleterious mutation effect, there is a distribution of deleterious mutation effects with sufficient density near zero”. The phenomena is conceptually understandable even without the model details; as mutations accumulate, additional mutations that would have previously been in the dangerous range can now be selected out, but other mutations whose effects previously had been too minor to be dangerous will now be in the dangerous range. Butcher concludes that “This contradicts previous work that indicated that epistasis will halt the ratchet”. This is further supported by Baumgardner et al. (2013), which shows that in simulations with biologically realistic parameters, synergistic epistasis does not halt genetic degeneration—but actually accelerates it. Likewise, Brewer et al. (2013), shows that related mechanisms (such as the mutation-count mechanism), fail to halt genetic degeneration.

The observation that modeling of the mutation selection process, using realistic parameters measured from nature, suggests that mutations should accumulate to the significant detriment of even large populations is a paradox highlighted by Kondrashov (1995):

I interpret the results in terms of the whole genome and show, in agreement with Tachida (1990), that VSDMs can cause too high a mutation load even when $N_e\approx 10^6-10^7$. After this, the data on the relevant parameters in nature is reviewed, showing that the conditions under which the load may be paradoxically high are quite realistic.

Kondrashov’s paradox suggests the critical need to better understand the mutation–selection process. Models show that deleterious mutations accumulate quickly in high mutation rate environments and small populations, and these models confirm observations from real organisms. These models further suggest that deleterious mutations will accumulate even in large populations with realistic parameters. The most complete models—those that like Butcher’s model use a distribution of mutational effects—suggest that the deleterious mutation accumulation is robust. There is a critical need for more complete models of mutation–selection and the surrounding biological parameters.

### Conceptual selection models

In this section we describe some conceptual selection models. These models are not first-principle models, being more conceptual than derived from competing / reproducing organisms.

Truncation selection is an artificial selection process wherein every generation the members of a population are sorted and ranked based upon a specific criteria (such as total fitness), then a threshold of performance is defined, and all individuals below this threshold are unconditionally eliminated. This process is used in breeding plants and animals. While this is an effective method for amplifying desirable traits, there is no reason to believe that nature “ranks and truncates” (see Crow and Kimura 1979) populations. Moreover, while truncation selection is useful in breeding, it is only useful for amplifying a trait up to a limit; beyond that limit loss of genetic variance leads to diminishing returns and genetic pathologies.

Truncation selection can sometimes happen in nature; but only in isolated and largely artificial circumstances. For example, when a bacterial colony is exposed to an antibiotic, all cells dies except for the resistent ones. However, almost universally, total fitness is affected by many traits, many genetic factors, many selection factors, and many environmental effects. Therefore, truncation is not generally relevant in natural populations.

## Modeling natural selection with mutations

In this section, we present a system of equations for a population model which, unlike the model in Fisher’s Theorem, can be studied as a dynamical system extended over time and which allows for the inclusion of a continuous supply of new mutations. In comparison to the models described in Sect. 2, we use a state space for the possible genotypes similar to the quasispecies approach, allow organisms to reproduce at rates proportional to fitness as with the infinite population models, but as with the finite population models, our total population is not forced to stay at carrying capacity and the mutations can create new genetic varieties not present in the original population. The model can be treated as an infinite population model using the differential equation in Eq. (3.2), or as a finite population model when any subpopulation with less than one organism is rounded down. Thus, we use the first principles approach to modeling from the previous infinite population models, but incorporate the flexibility of the previous finite ones.

### Differential equations with mutations

For a given population, divide the population into a collection of *N* subpopulations $\{P_1,\ldots ,P_N\}$, where all organisms in subpopulation $P_i$ have the same fitness. This could be done by grouping them be genotype ($P_i$ being all organisms with genotype *i* as in the quasispecies model), but two genotypes with the same fitness will be indistinguishable in our model and do not need to be separated. Alternatively, we could divide the range of finesses for the whole population into *N* subintervals and define $P_i$ to be the organisms with fitness in the $i_{th}$ subinterval.

Denote the birth rate of the $i\mathrm{th}$ subpopulation by $b_i$ and the death rate by $d_i$, with resulting net intrinsic growth rate of $m_i=(b_i-d_i)$. Suppose that, in addition, the probability that the progeny of a parent with fitness $m_j$ has fitness $m_i$ is given by a probability distribution function $f_{ij}$. It is assumed that this the distribution is a function of $(m_i-m_j)$; that is, it depends on the difference in fitness from parent to offspring and not on the particular genotype of the parent. Describing this distribution, either in general terms or with a class of formulas, is an ongoing research endeavor, although it is known that the distribution is highly skewed in favor of deleterious mutations [See Ohta (1977), Kimura (1979) and further discussion in Sect. 2].

Ignoring mutations for the moment and considering only the fitness, which is assumed equal to the Malthusian growth rate, yields a system consisting of one exponential growth model for each subpopulation:

3.1 $$\begin{aligned} \frac{\text {d}P_i}{\text {d}t} = m_i P_i. \end{aligned}$$

This is the system used for the special example of Fisher’s theorem (Crow and Kimura 1970, p. 10). To derive our equations including the effect of mutations, we can rewrite Eq. (3.1) using $m_i=b_i-d_i$ to get

$$\begin{aligned} \frac{\text {d}P_i}{\text {d}t} = b_i P_i - d_i P_i. \end{aligned}$$

In this equation, the first term is the rate at which organisms in the $i\text {th}$ subpopulation are born and the second term is the rate at which these organisms die. To incorporate mutations, we need to consider not just births of subpopulation *i* from within this subpopulation, but the total births of organisms $P_i$ resulting from parents of all subpopulations. Because, $b_jP_j$ is the rate at which the population of $P_j$ is giving birth and $f_{ij}$ is the fraction of these births that are in $P_i$, the rate at which progeny are born in $P_i$ from parents within $P_j$ is $b_j f_{ij} P_j$, and thus the rate at which organisms of subpopulation *i* are born from parents of all subpopulations is $\sum _j b_j f_{ij} P_j$. The governing equation for our model system is then:

3.2 $$\begin{aligned} \frac{\text {d}P_i}{\text {d}t} = \sum _j b_j f_{ij} P_j - d_i P_i. \end{aligned}$$

Observe that if we remove the effect of mutations ($f_{ij} = \delta _{ij}$, where $\delta _{ij} = 1$ if $i=j$ and is zero otherwise), then we retrieve the system in Eq. (3.1).

### The fundamental theorem of natural selection with mutations

In this section we present the main theorem of the paper, proving a formula for the rate of change of the average fitness of a population based on the variance in fitness and the probability distribution for the effect of mutations on fitness.

#### Theorem 2

The fundamental theorem of natural selection with mutations

The rate of change of mean fitness $\bar{m}=(\sum _i P_i m_i)/\sum _i P_i$ of a population is given by

3.3 $$\begin{aligned} \frac{\mathrm{d} \bar{m}}{\mathrm{d} t} = \text {Var}(m) + \frac{1}{\sum _i P_i} \sum _i\left( \left( \sum _jb_jf_{ij}P_j\right) -b_iP_i \right) (m_i-\bar{m}). \end{aligned}$$

If we let $B_i^\text {in}$ be the rate at which organisms of genotype *i* are born, and let $B_i^\text {out}$ be the rate at which they are giving birth, then the equation becomes

3.4 $$\begin{aligned} \frac{\mathrm{d} \bar{m}}{\mathrm{d} t} = \text {Var}(m) + \frac{1}{\sum _i P_i} \sum _i\left( B_i^\text {in}-B_i^\text {out} \right) (m_i-\bar{m}). \end{aligned}$$

Observe that, as before, if we remove the effect of mutations (by setting $f_{ij}=\delta _{ij}$) then we retrieve the special case of Fisher’s fundamental theorem of natural selection from  Crow and Kimura (1970). Also observe that the right-hand most term represents a downward (negative-valued) pressure when births tend to decrease fitness. A better understanding of this new term would be extremely valuable to understanding Theorem 2.

#### Proof

By the quotient rule applied to $\bar{m}=(\sum _i P_i m_i)/\sum _i P_i$,

$$\begin{aligned} \frac{\text {d}\bar{m}}{\text {d}t} = \frac{ \left( \sum _i P_i' m_i\right) \left( \sum _iP_i\right) - \left( \sum _i P_i'\right) \left( \sum _iP_im_i\right) }{ \left( \sum _i P_i\right) ^2} \end{aligned}$$

Separating the fraction, using $\bar{m}=(\sum _i P_i')/\sum _i P_i$), and then recombining gives

3.5 $$\begin{aligned}&= \frac{ \sum _i P_i' m_i }{ \sum _i P_i } - \frac{\left( \sum _i P_i'\right) \left( \sum _iP_im_i\right) }{ \left( \sum _i P_i\right) ^2 } \nonumber \\&= \frac{ \sum _i P_i' m_i }{ \sum _i P_i } - \frac{ \bar{m} \sum _i P_i' }{ \sum _i P_i } \nonumber \\&= \frac{ \sum _i P_i' m_i - \bar{m} \sum _i P_i' }{ \sum _i P_i } \nonumber \\&= \frac{ \sum _i P_i'(m_i - \bar{m}) }{ \sum _i P_i }. \nonumber \\ \end{aligned}$$

Now using the definition of $P_i'$ gives

3.6 $$\begin{aligned}&= \frac{ \sum _i \left( \sum _j P_j b_j f_{ij} - d_i P_i\right) \left( m_i - \bar{m}\right) }{ \sum _i P_i }. \nonumber \\ \end{aligned}$$

Solving $m_i = b_i-d_i$ for $-d_i$ and substituting, and then adding $0 = \bar{m}P_i - \bar{m}P_i$ gives

3.7 $$\begin{aligned}&= \frac{ \sum _i \left( \sum _j P_j b_j f_{ij} - b_i P_i + m_iP_i + \bar{m}P_i - \bar{m}P_i\right) \left( m_i - \bar{m}\right) }{ \sum _i P_i }. \nonumber \\ \end{aligned}$$

Separating the finite sum yields

3.8 $$\begin{aligned}&= \frac{ \sum _i \left( \sum _j P_j b_j f_{ij} - b_i P_i\right) \left( m_i - \bar{m}\right) + \sum _i \bar{m}P_i\left( m_i - \bar{m}\right) + \sum _i P_i\left( m_i - \bar{m}\right) \left( m_i - \bar{m}\right) }{ \sum _i P_i }. \nonumber \\ \end{aligned}$$

Seperating the sum across the top gives

3.9 $$\begin{aligned}&= \frac{ \sum _i \left( \sum _j P_j b_j f_{ij} - b_i P_i\right) (m_i - \bar{m})}{ \sum _i P_i }+ & {} \frac{\sum _i \bar{m}P_i(m_i - \bar{m})}{ \sum _i P_i }+ & {} \frac{\sum _i P_i(m_i - \bar{m})(m_i - \bar{m}) }{ \sum _i P_i }. \nonumber \\&= \frac{ \sum _i \left( \sum _j P_j b_j f_{ij} - b_i P_i\right) (m_i - \bar{m})}{ \sum _i P_i }+ & {} 0+ & {} \text {Var}(m), \nonumber \\ \end{aligned}$$

which completes the proof.

## Analytic solutions

In this section we provide analytical results for the main equation, Eq. (3.2) when there are a finite number of fitness levels and the population is assumed infinite. In this case, the differential equation is solvable like many of the models presented in Sect. 2.2. While the focus of this paper is the derivation and analysis of this equation along the lines of Fisher’s work and analysis of fitness distributions using realistic biological parameters, it is useful to consider related solvable systems.

Using the notation from Eq. (3.2), define the matrix *W* by

$$\begin{aligned} w_{ij} = b_j f_{ij} - d_i \delta _{ij}. \end{aligned}$$

Note that we are making an implicit assumption here that there are a finite number of fitness levels. Equation 3.2 can then be rewritten as

$$\begin{aligned} \mathbf {P}'=W\mathbf {P}. \end{aligned}$$

This is a linear differential equation. The sole equilibrium solution is the $\mathbf {P}=\mathbf {0}$, and all other solutions will be asymptotic to the eigenvector corresponding to the largest eigenvalue of *W*.

By treating the system as a differential equation, we are implicitly assuming an infinite population; in this model, each $P_i$ will be non-zero positive, but can be arbitrarily small. The system given in Equation 4 is analogous to a quasispecies model discussed in Sect. 2.2. The difference is that in a quasispecies each $P_i$ is the number of organisms with the $i\mathrm{th}$ genotype whereas in Equations 4 and 3.2, $P_i$ is the number of organisms with fitness in the $i\mathrm{th}$ fitness level. These systems are considered infinite populations in part because each subpopulation $P_i$ remains non-zero, regardless of how small $P_i$ is compared to the total population. In the numerical simulations of Sect. 5, we address this be setting any subpopulation with sufficiently small fraction of the total population to zero.

Even with the infinite population nature, quasispecies analysis is consistent with our results. The error threshold is the mutation rate separating adaptation (the population distribution with most mass on high fitness genotypes) from failure to adapt (population distribution spread across to include lower fitness genotypes), and Theorem 2 gives a condition on the population and mutation distribution separating increasing mean fitness from decreasing mean fitness.

This paper focuses on the implications of the main system as it relates to Fisher’s work using biological realistic parameters. Even in an infinite population, the mean fitness goes to equilibrium fitness, and not perpetual increase as Fisher predicted. Beyond this, we will not develop the infinite population approach further. However, there are interesting questions of what quasispecies type analysis would imply for Equation 4, where we a considering mutations between fitness levels with probabilities prescribed by known fitness effects distributions. For example, under what mutation effect distributions will the resulting eigenvector have positive or negative mean fitness, or how does the behavior of the solution to the linear system compare to solutions when finite populations are considered.

## Numerical simulations

In this section we present numerical results for the main system and plot components of the resulting numerical solution to illustrate Theorem 2. All plots in this section were created using the online JavaScript page developed for modelling this system (Basener 2013a).

Because the focus of this paper is on implications of the system for biological populations, we make a modification of Eq. (3.2) that effectively restricts to finite-sized populations. To remain biologically realistic, we assume a finite population: any subpopulation $P_i$ that contains less than some fraction of the population is assumed to contain zero organisms. For the numerical simulations, we set $P_i=0$ whenever $P_i$ is less than $10^{-9}$ of the total population. This approximates a total population of $10^9$ and eliminates any subpopulation with less than a single organism. The only case where this made an observable difference was Sect. 5.4. In that case, without the finite-population condition subpopulations remain viable even when they contain less than a fraction of an organism. As a result, extremely small, biologically nonsensical, populations control the observed results and obscure the effect of mutations on the population as a whole.

We assume for all simulations that the initial population has a Gaussian fitness distribution with mean of $\bar{m}=0.044$ (as is commonly seen in a typical pre-industrial human population) and a standard deviation of $\sigma =0.005$. Also in all simulations we model the fitness levels in the population, measured in Malthusian growth rate, over 500 evenly spaced discrete values ranging from $m = -\,0.05$ to $m = 0.15$. The death rate is set to $d_i = 0.1$ (equivalently, the populations dies at $10\%$ per year) and the birth rate is determined for each *i* using $b_i = m_i + d_i$. We also assume that the individuals within the initial population have fitness level ranges up to 0.1, which includes values up to 11.2 standard deviations above the mean. While there will always be some upper limit of fitness within the organisms of any population, we chose this specific value to permit extremely high fitness values and for the convenience of display in the plots. The actual maximal value is not important.

As discussed previously, Fisher assumed that large magnitude deleterious mutations would be eliminated by selection, and the remaining small magnitude mutations would be neutral with an average change in fitness of zero. There is no support for “selecting out” mutations before incorporating them into the mutation–selection model; if they get selected out it has to take place within the competition between populations in the model as in real biological populations. Recent research demonstrates that the effect of mutations are highly skewed to being deleterious, with most mutations having a very slight deleterious effect on fitness.

The distribution can be modeled using a Gamma probability distribution (Kimura 1979).

$$\begin{aligned} f(s') = \alpha ^\beta e^{-\alpha s'}s'^{\beta -1}/\varGamma (\beta ), \end{aligned}$$

where $s'$ is the change in fitness (in absolute value), $f(s')$ is the probability for this change in fitness to occur from parent to child, $\alpha =\beta /\bar{s}'$ where $\bar{s}'$ is the mean selective disadvantage, and $\beta$ is a parameter, called either the shape parameter or rate parameter, with $0<\beta \le 1$. Kimura suggests that a typical value for $\bar{s}'$ is $10^{-3}$ for deleterious mutations, which is the value we use.

The parameter $\beta$ determines the tendency for mutations to be neutral; in the limiting case as $\beta \rightarrow 0$, all mutations are neutral and in the case with $\beta = 1$, we regain Ohata’s model (Ohta 1977) in which there are not a sufficient number of nearly-neutral mutaitons, according to Kimura (1979). We choose $\beta =0.5$ which is the estimation given by Kimura (1979).

$$\begin{aligned} f(s') = 500^{0.5} e^{-500 s'}s'^{-0.5}/\varGamma (0.5) \approx 12.6 e^{-500 s'} s'^{-0.99} \end{aligned}$$

If we use $\triangle _{i,j}$ to denote the change in fitness from $m_i$ to $m_j$ then our formula becomes

5.1 $$\begin{aligned} f_{i,j} \approx 12.6 e^{-500\triangle _{i,j}} {\triangle _{i,j}}^{-0.5} \end{aligned}$$

This distribution used by Kimura only accounts for deleterious mutations. He points out: “Note that in this formulation, we disregard beneficial mutants, and restrict our consideration only to deleterious and neutral mutations. Admittedly, this is an oversimplification, but as I shall show later, a model assuming that beneficial mutations also arise at a constant rate independent of environmental changes leads to unrealistic results” (Kimura 1979).

**Fig. 1:**
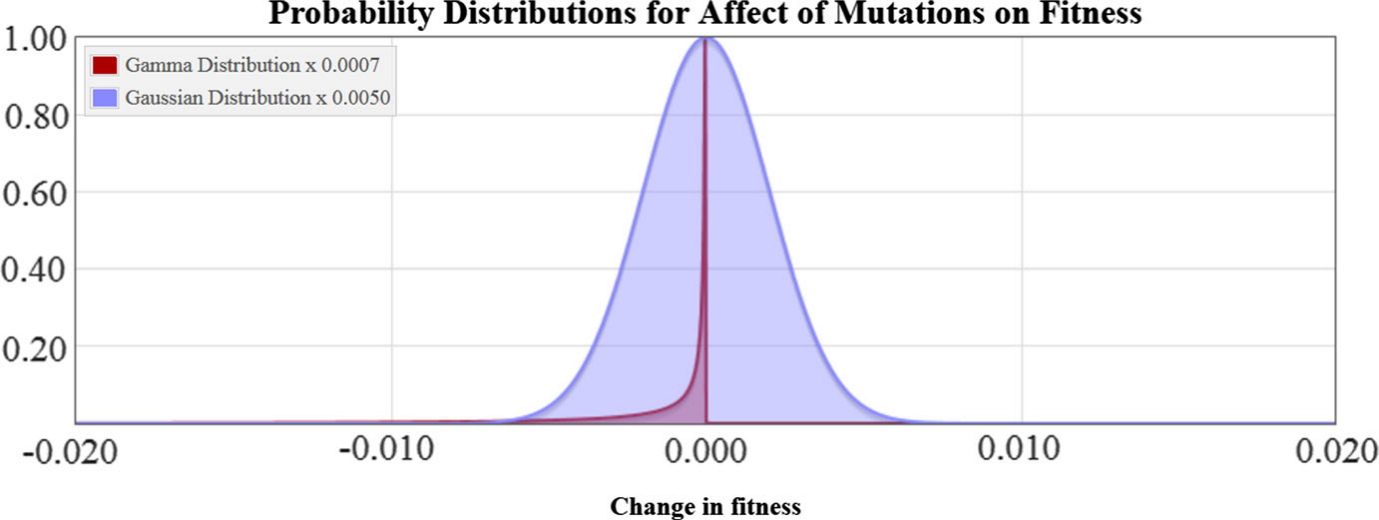
The Gaussian and Gamma probability distributions for the effect of mutations on fitness. Note that the distributions have been scaled different for this plot so that the mode of both is equal to one, as noted in the Figure Key. The Gamma distribution requires that nearly all mutations have very small deleterious fitness effects

For our purposes of dynamically modeling the change in fitness over time, we require some beneficial mutations (Otherwise long-term fitness increase would be impossible). We make the simplifying assumption that the fraction of beneficial mutations is 0.001, and we also assume that the distributions for beneficial and deleterious mutations otherwise have the same parameterization. This fraction of beneficial mutations overestimates the actual rate of beneficial mutations by orders of magnitude. Gerrish and Lenski estimate the ratio of deleterious to beneficial mutations at $10^6:1$ Gerrish and Lenski (1998b). Other estimates indicate that the number of beneficial mutations is too low to be measured statistically (Ohta 1977; Kimura 1979; Elena et al. 1998; Gerrish and Lenski 1998a). By assuming the same parameterization for beneficial and deleterious mutations, we also overestimate the range of the beneficial mutations because beneficial mutations tend to consistently have more modest fitness effects. A plot of the distribution for the effect of mutations is shown in Fig. 1, showing both the symmetric Gaussian distribution imagined by Fisher and the Gamma distribution we use building from Kimura’s work. Note that beneficial mutations are present in the Gamma distribution but are too unlikely to appear on this plot.

### Simulation with no mutations and a short time-span

First we present a simulation that demonstrates Fisher’s Theorem in its original form by modeling a population over a short time period, wherein no new mutations are arising. We have to use a short time period because his theorem only gives a formula for the rate of change of fitness at one instant. We can approximate this instantaneous rate of change by only running our model for a short time.

As mentioned above, we assume an initial fitness distribution with mean equaling 0.044 and a standard deviation equaling 0.005 (Recall also that the upper limit on the fitness of organisms in the initial distribution is $m=0.1$).

**Fig. 2:**
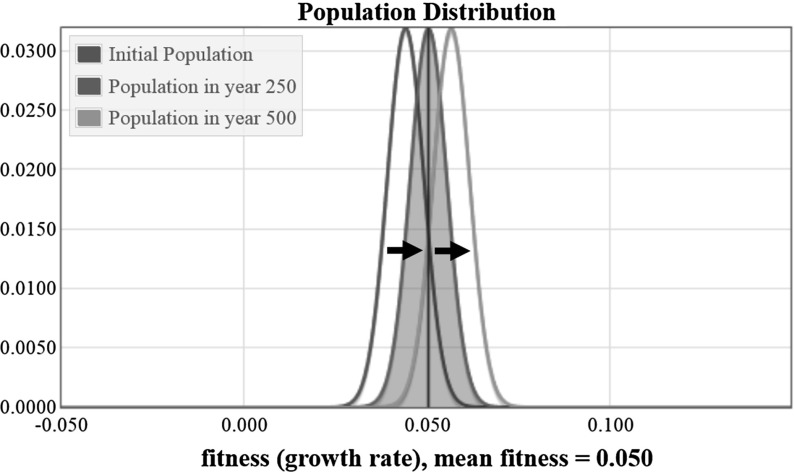
Plots showing the change in the population distribution from year zero to year 500

The solution to the set of equations in Eq. (3.2) is a population distribution that changes over time. If we plot the results of a numerical simulation of Eq. (3.2) over a short time interval without mutations, then we get as expected a population distribution that has a steadily increasing mean fitness. The steady increase in the population can be observed in the plot shown in Fig. 2, which shows the initial population fitness distribution, the distribution in the year 250, and the distribution at the end of the simulation in year 500 (We use years here because our growth rate factor *m* is defined in terms of years).

**Fig. 3:**
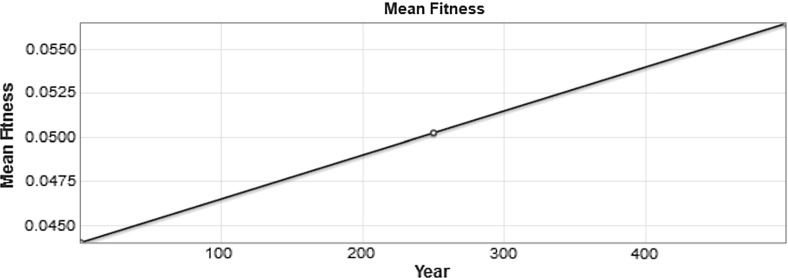
The value of the mean population fitness as a function of time is shown. The value in year 250 is marked with a blue circle (color figure online)

The value of the mean fitness of the population is plotted in Fig. 3 as a function of time, for the system with no mutations. The variance for our initial distribution is $\sigma ^2 = 0.005^2 = 0.000025$. The mean fitness shown in Fig. 3 increases by approximately 0.0025 every 100 years—that is, the slope of the line is $\triangle y / \triangle x = 0.0025/100 = 0.000025$. Thus, as expected, we observe the mean fitness increasing at a constant rate approximately equal to the variance. Not shown is the change in variance over time, which is negligible.

The numerical example in the above section illustrates Fisher’s fundamental theorem of natural selection. The higher fitness organisms reproduce more quickly, and thus come to dominate the population resulting in increasing mean fitness; specifically the rate of increase in fitness is proportional to the variance. Because Fisher’s Theorem only applies to an instantaneous rate of change in fitness of the population, and not to the change in the population over time, it is important that we kept this model restricted to a small time interval. It is interesting in this example that the population distribution appears to simply be translating to the right, with no change in the shape of the distribution. This suggests the conjecture that the second derivative of the mean fitness is equal to zero if the fitness distribution of the initial population is Gaussian.

### Simulation with no mutations and a long time-span

**Fig. 4:**
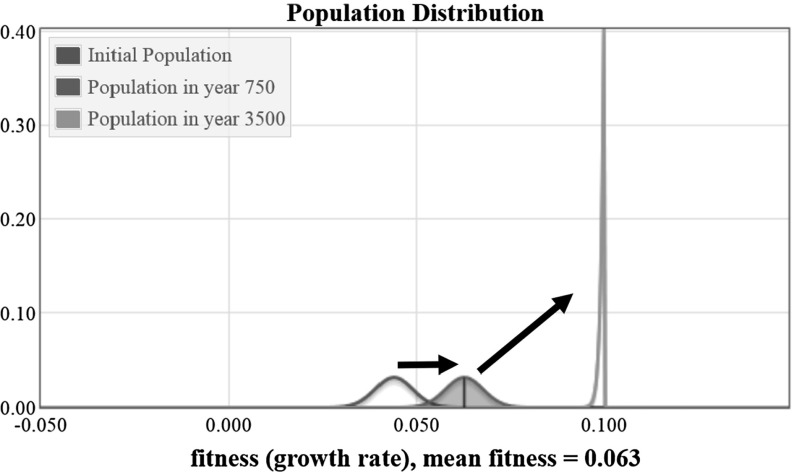
Plots showing the change in the population distribution from year zero to year 3500

We present a simulation that demonstrates the limitations of Fisher’s Theorem applied to a population changing over time by modeling a population with no mutations over a longer time period. By using the same system as in Sect. 5.1 but with a time period of 3500 years, we see the population increase in fitness until it runs up against the maximal fitness of the initial population, which is $m=0.1$. The population distributions resulting from this system are shown in Fig. 4. Observe that there is the initial population with mean fitness of 0.044, then a transitional population distribution from year 750 with mean fitness of 0.063, and the final population distribution running up against the maximal fitness of the population.

**Fig. 5:**
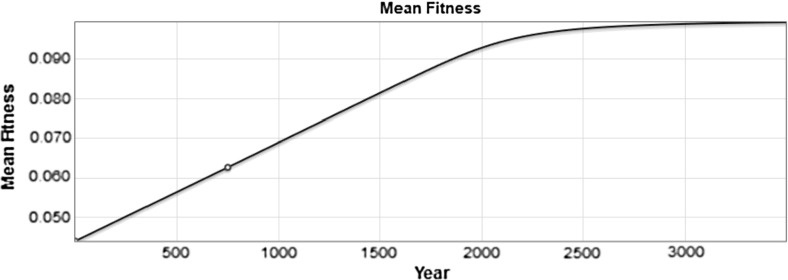
The value of the mean population fitness as a function of time is shown. The value in year 750 is marked with a blue circle (color figure online)

The value of the mean fitness of the population is plotted as a function of time in Fig. 5. The mean fitness initially increases at a constant rate approximately equal to the variance and then levels off as it approaches the upper limit of $m=0.1$. As this limit is approached, the distribution loses variance, becoming taller and narrower in shape (See Fig. 4). As a result the increase in fitness slows down. Over a longer time period, the fitness approaches the limiting value of $m=0.1$.

**Fig. 6:**
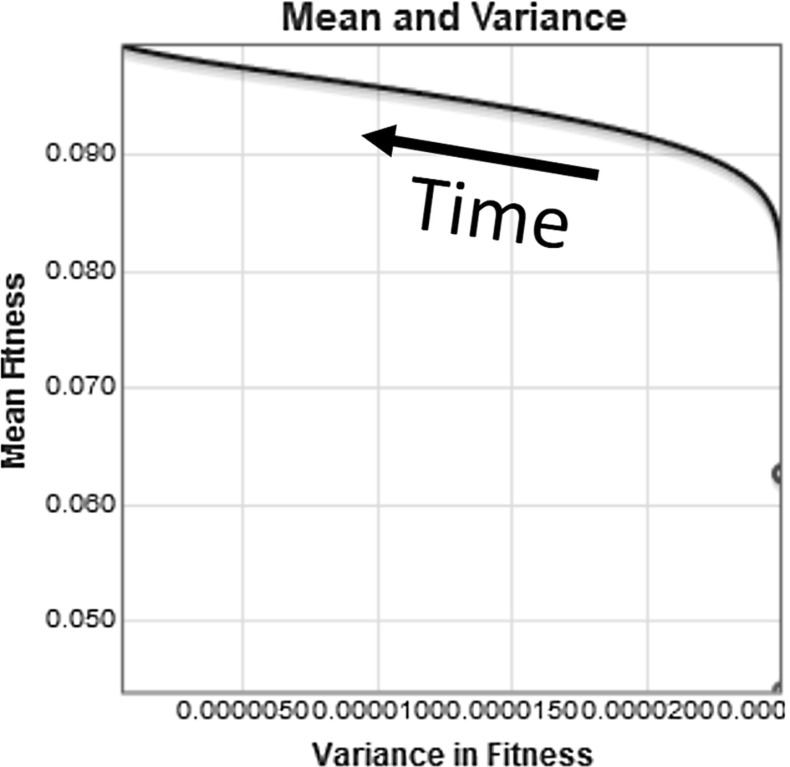
Plotting the mean fitness and the variance, we see the increase in fitness up to its limiting value of 0.1 and the eventual decline in variance

The plot of the mean fitness and variance of the population for this numerical simulation is shown in Fig. 6. In this plot we see the mean fitness increasing at first and then approaching the limit of $m=0.1$ while the variance is initially steady and then decreases toward its limiting value of $var = 0$.

The numerical simulations in this section illustrate the effects of selection apart from mutation. In this case we have an initial increase in fitness until the variance is consumed in the optimization process and the population fitness approaches its limiting maximal value. This is what is observed by selective plant and animal breeders. Selective breeding within a genetically diverse population results in an initial increase in some predetermined trait, for example height. But selective progress always eventually slows down and the trait approaches a natural limit.

### Simulation with a Gaussian distribution for mutational effects

**Fig. 7:**
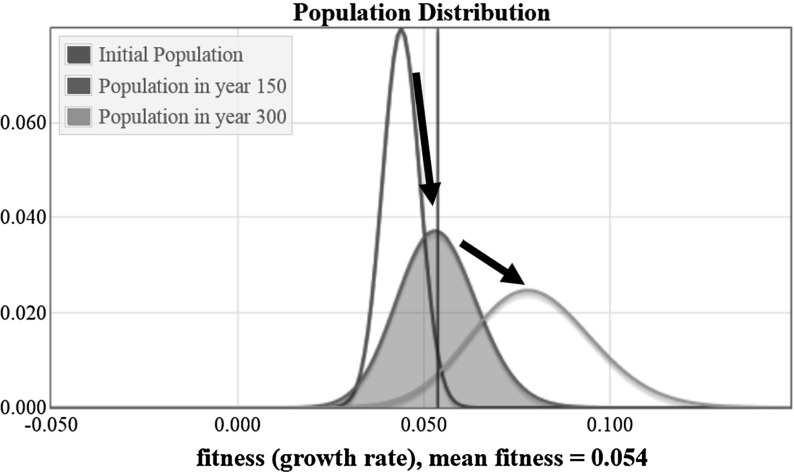
Plots showing the change in the population distribution from year zero to year 500 for a system with a Gaussian distribution for the effect of mutations

Fisher assumed that the only mutations that needed consideration in a mathematical model are those with a mutational effect with equal probability of being beneficial as deleterious. A numerical solution to the set of equations in Eq. (3.2), assuming a Gaussian distribution (mean equal to zero and standard deviation equal to 0.002) is shown in Fig. 7.

**Fig. 8:**
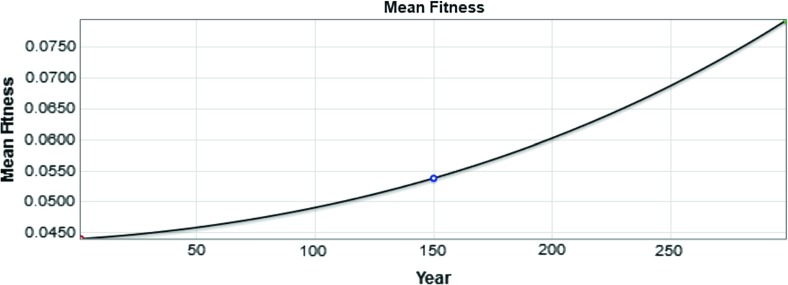
The value of the mean population fitness as a function of time is shown. The value in year 150 is marked with a blue circle (color figure online)

It is clear from Fig. 7 that the mean fitness of the population is increasing, as Fisher expected. Also, in this example it appears that the population distribution has an increasing variance as time passes. It takes less than 500 years for this population to reach a mean fitness of 0.1, whereas the numerical solution in Sect. 5.2 was run for 3500 years without reaching this fitness value. This shows the profound effect of modeling a mutation distribution having a zero net change in fitness. The value of the mean fitness of the population is plotted in Fig. 8 as a function of time.

**Fig. 9:**
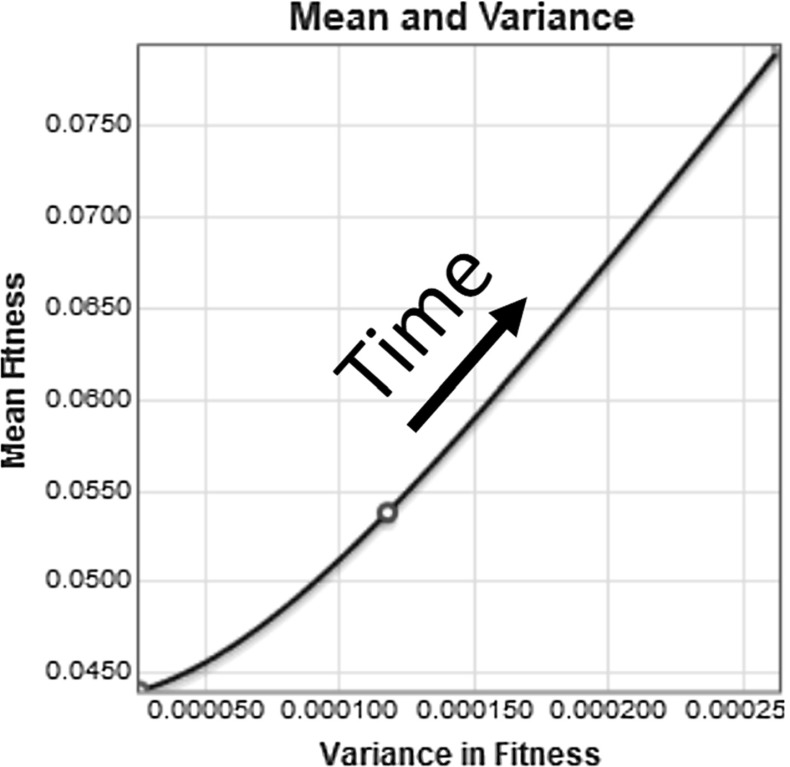
Plotting the mean fitness and the variance, we see that both values are increasing as time passes

The plot of the mean fitness and variance of the population for this numerical simulation is shown in Fig. 9. In this plot we see that both the mean fitness and the variance are increasing with time. The increasing variance corresponds to the increasing rate of change of fitness, observable in the concave up shape of the graph in Fig. 8.

The solution in this section indicates an increasing rate of change in mean fitness. Not only is the mean fitness increasing, but it is accelerating. In contrast, the case with no mutations in Sect. 5.1 had a mean fitness that increased at a constant rate. It is possible that for an initial population with a Gaussian distribution and with Gaussian mutations, the mutations will cause a continually increasing fitness and variance. Although this system is biologically unrealistic, it behaves mathematically as Fisher would have expected.

**Fig. 10:**
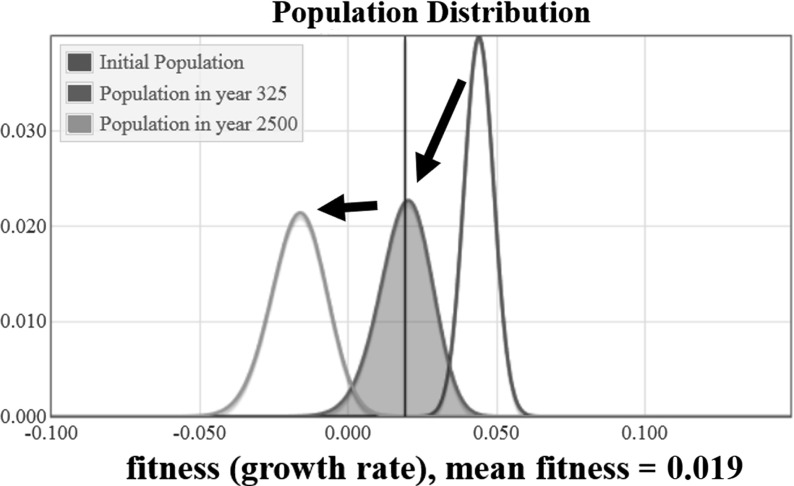
Plots showing the change in the population distribution from year zero to year 2500

### Simulation with a gamma distribution for mutational effects

The results of a numerical solution to Eq. (3.2) using the Gamma distribution from Eq. (5.1) for mutation effects on fitness and with the finite population condition are shown in Fig. 10. It is worth noting that without the finite-population condition described at the beginning of this section, which requires that all subpopulations with less than a single organism be set to zero, some numerical solutions went to an equilibrium. The focus of this paper is on biological implications of the system, so we only show results for the system with the finite-population condition.

The initial population distribution has a mean of 0.044, shown in red. The final equilibrium population has a mean fitness a little greater than $-0.014$ , shown in green. Negative fitness means deaths exceed births, such that the population will shrink regardless of resources. There is a transient state distribution shown in blue, which is slightly bimodal and has a mean fitness of 0.019.

**Fig. 11:**
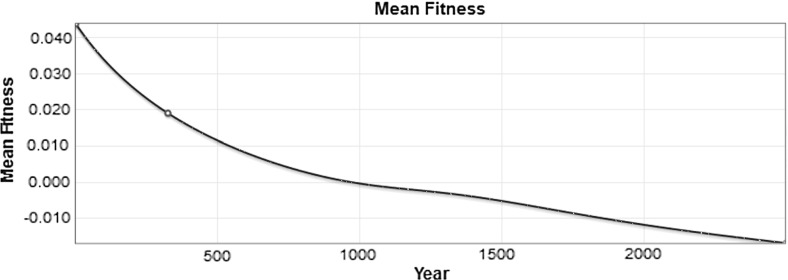
The value of the mean population fitness as a function of time is shown. The value in year 325 is marked with a blue circle (color figure online)

The mean fitness is plotted as a function of time in Fig. 11. We see that the fitness decreases steadily to about ${-}$ 0.014 over 2500 years. Moreover, the fitness appears to be continuing to fall without-bound.

**Fig. 12:**
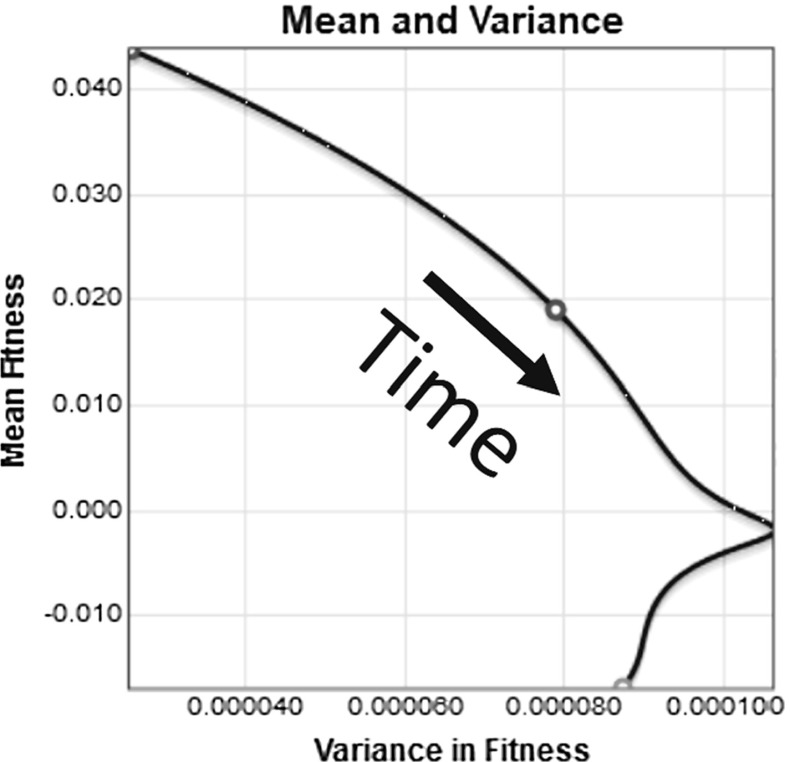
Plotting the mean fitness and the variance, we see that the fitness decreases steadily with the variance increases, then falls. The mean fitness becomes negative, deaths exceed births—causing population collapse

The mean fitness and variance are plotted in Fig. 12. This plot suggests that the fitness is decreasing steadily, but the variance increases, goes through a cusp, and then decreases (as the population collapses). This cusp occurs about the point where the mean fitness (growth rate) is zero. This is the point where the third and final stage of Lynch’s mutational meltdown model begins. While our population is held at $10^9$, we still see the continuing decline in fitness. If the total population size were variable, it is likely that we would see compounding decline of a mutational meltdown.

In this section we have observed that for a finite population, having what we consider to be a realistic distribution of mutational effects, the mutation–selection process results in continuous fitness decline. Our parameter vales are very generous, overestimating the number and magnitude of beneficial mutations and with a population size of $10^9$, making this simulation a best-case scenario for improving fitness. In terms of Theorem 2, the downward pressure from mutations overwhelms the upward pressure of selection. We observe that it problematic to define parameter settings that are biologically realistic yet result in continuous fitness increase, supporting the modeled buildup of very slightly deleterious mutations described in Kondrashov’s paradox.

## Discussion

Arguably, R.A. Fisher was the singular founder of the field of population genetics. His book, The Genetical Theory of Natural Selection, established for the first time the connection between genetics and natural selection. Within that pioneering book, Fisher presented his famous fundamental theorem of natural selection.

The value of Fisher’s many mathematical contributions to the biological sciences cannot be overstated. However, because he was a pioneer who lived before the biological revolution, Fisher’s understanding of mutations was very limited, and his formulation of his famous theorem was less than ideal. His formulation was cumbersome and was not suited to dynamical analysis. More importantly, his formulation did not allow the modeling of newly arising mutations, which was pivotal to his general line of reasoning. It is for this reason that we have re-formulated his theorem - so it can be analyzed dynamically and can incorporate continuously arising new mutations.

When we dynamically analyzed Fisher’s theorem as he originally presented it (i.e., apart for newly arising mutations), we observed what should be logically obvious. When starting with pre-existing genetic variants within the population (both beneficial and deleterious allelic variants), we saw that natural selection favored the beneficial variants over the deleterious variants, resulting in fitness increase (see Sect. 5.1). The more abundant were the initial genetic variants, the more rapid was the fitness increase. We further observed (and it should be equally obvious) that as selection eliminated the deleterious variants and amplified the beneficial variants to fixation, genetic variation moved toward zero and consequently selection became ineffective and fitness soon stopped increasing (see Sect. 5.2). This was observed with both Fisher’s original formation and with our improved formulation.

This result may be surprising for many people who have been taught that Fisher’s Theorem guarantees unlimited fitness increase. Apart from continuously arising new mutations, Fisher’s theorem only yields a brief period of genetic enhancement based upon sorting through the pre-existing allelic variants. The population then quickly approaches a natural limit and becomes static. This stasis is a fundamental element of the formulation in Fisher’s Theorem (apart from Fisher’s unstated corollary, which assumes a continuous supply of newly arising mutations).

When we modeled Fisher’s Corollary by adding continuously arising new mutations that have a symmetrical distribution of mutational fitness effects, we observed very different results. Fitness increased rapidly and continuously. When mutation rate was high, mutations accumulated faster than they went to fixation, resulting in an on-going increase in genetic variance and a subsequent fitness increase that was accelerating (see Sect. 5.3). This is much closer to Fisher’s original expectations.

However, through no fault of his own, Fisher did not know the molecular nature of mutations and incorrectly assumed that mutational effects (positive and negative), would be effectively symmetrical and balanced. This assumption was profoundly incorrect, and so the results seen when using a symmetrical mutation distribution have no correspondence with biological reality. Because the premise underlying Fisher’s corollary is now recognized to be entirely wrong, Fisher’s corollary is falsified. Consequently, Fisher’s belief that he had developed a mathematical proof that fitness must always increase is also falsified.

We next modeled Fisher’s theorem with the newly arising mutations having a more realistic distribution. For both the bad and the good mutations, we employed the same gamma probability distribution, but we used a deleterious:beneficial ratio of 1000:1. This is a very generous ratio, in light of many studies (see Gibson et al. (2013); Sanford et al. (2013); Nelson and Sanford (2013); Montanez et al. (2013)). The result was that fitness declined continuously. Malthusian fitness declined to the point of going below zero, meaning that the population was shrinking continuously regardless of the carrying capacity of the environment. The net affect of the new mutations was very consistently deleterious, and the upward pressure on fitness due to natural selection was not sufficient to reverse the on-going mutational degeneration.

What we have discovered is that, contrary to Fisher’s claim, continuously increasing population fitness is not an inherent property of life. Mutations by themselves drive fitness down. Natural selection may or may not be able to reverse this genetic degeneration. There are a large number of biological variables that determine whether the fitness of a population will increase or decrease. For example, there are important variables associated with the population itself (is it diploid? is it sexual? effective population size?). Likewise, there are important variables associated with the mutations (mutation rate? mutation distribution? degree of dominance?). There are important variables associated with how mutations interact (is there dominance? is there epistasis? is there linkage?). There are important variables associated with the selection process (proportion of offspring selectively eliminated? natural probability selection or artificial truncation selection? how does environmental noise affect fitness heritability? does selection for many mutations simultaneously result in selection interference?).

All these variables combine to determine if a population’s fitness will increase or decrease. Unfortunately, there is no simple mathematical formula that can simultaneously account for all these variables simultaneously. As a general rule, the simplifying assumptions as are required for a purely mathematical approach to population genetics force researchers to ignore many variables that tend to reduce the efficiency of selection. This can result in overly optimistic expectations regarding the net fitness effect of the downward pressure of mutations versus upward pressure of selection. Arguably, the only way to account for the many biological variables that simultaneously affect the mutation–selection process is by using comprehensive numerical simulations (See Sanford et al. 2007a, b; Nelson and Sanford 2013). As numerical simulations become more comprehensive (hence more realistic), net gain in fitness seems to become increasingly problematic (See Sanford et al. 2007b; Carter and Sanford 2012; Gibson et al. 2013; Sanford et al. 2013; Nelson and Sanford 2013), consistent with the results of this paper.

Apart from theoretical and mathematical reasons for doubting the biological validity of Fisher’s central thesis (that fitness always increases), there is now also abundant empirical evidence against his thesis. For example, ecological observations consistently show that Fisher’s thesis is not true, and that as a general rule a natural population’s fitness is static. Essentially all natural populations have substantial genetic variance, yet most such populations do not show continuously increasing fitness. This is due to very low fitness heritability, associated with high levels of environmental noise (See Merila and Sheldon 2000; Kruuk et al. 2000, 2002). Furthermore, extinctions and near extinctions happen all the time, which are clearly antithetical to Fisher’s thesis. In addition, the genetic degeneration of certain organisms has been recorded within historical time frames (Carter and Sanford 2012). Lastly, many population geneticists have expressed grave concerns regarding possible conditions where human mutational degeneration overwhelms the stabilizing effect of natural selection (See Lynch 2016). Further research is needed to help us understand exactly what biological conditions are required to ensure a population’s fitness stability.

## Conclusions

We have re-examined Fisher’s fundamental theorem of natural selection, focusing on the role of new mutations and consequent implications for real biological populations. Fisher’s primary thesis was that genetic variation and natural selection work together in a fundamental way that ensures that natural populations will always increase in fitness. Fisher considered his theorem to essentially be a mathematical proof of Darwinian evolution, and he likened it to a natural law. Our analysis shows that Fisher’s primary thesis (universal and continuous fitness increase) is not correct. This is because he did not include new mutations as part of his mathematical formulation, and because his informal corollary rested upon an assumption that is now known to be false.

We have shown that Fisher’s Theorem, as formally defined by Fisher himself, is actually antithetical to his general thesis. Apart from new mutations, Fisher’s Theorem simply optimizes pre-existing allelic fitness variance leading to stasis. Fisher realized he needed newly arising mutations for his theorem to support his thesis, but he did not incorporate mutations into his mathematical model. Fisher only accounted for new mutations using informal thought experiments. In order to analyze Fisher’s Theorem we found it necessary to define the informal mutational element of his work as Fisher’s Corollary, which was never actually proven. We show that while Fisher’s Theorem is true, his Corollary is false.

In this paper we have derived an improved mutation–selection model that builds upon the foundational model of Fisher, as well as on other post-Fisher models. We have proven a new theorem that is an extension of Fisher’s fundamental theorem of natural selection. This new theorem enables the incorporation of newly arising mutations into Fisher’s Theorem. We refer to this expanded theorem as “The fundamental theorem of natural selection with mutations”.

After we re-formulated Fisher’s model, allowing for dynamical analysis and permitting the incorporation of newly arising mutations, we subsequently did a series of dynamical simulations involving large but finite populations. We tested the following variables over time: (a) populations without new mutations; (b) populations with mutations that have a symmetrical distribution of fitness effects; and (c) populations with mutations that have a more realistic distribution of mutational effects (with most mutations being deleterious). Our simulations show that; (a) apart from new mutations, the population rapidly moves toward stasis; (b) with symmetrical mutations, the population undergoes rapid and continuous fitness increase; and (c) with a more realistic distribution of mutations the population often undergoes perpetual fitness decline.

In the light of Fisher’s work, and the problems associated with it, we also examined post-Fisher models of the mutation–selection process. In the case of infinite population models, what has commonly been observed is that populations routinely go to equilibrium or a limit set—such as a periodic orbit. They do not show perpetual increase or decline in fitness, but are restricted from either behavior because of the model structure (an infinite population with mutations only occurring between pre-existing genetic varieties). On a practical level, all biological populations are finite. In the case of finite population models, the focus has been upon measuring mutation accumulation, as affected by selection. Finite models clearly show that natural populations can either increase or decrease in fitness, depending on many variables. Not only do other finite mathematical population models show that fitness can decrease—they often show that only a narrow range of parameters can actually prevent fitness decline. This is consistent with very many numerical simulation experiments, numerous mutation accumulation experiments, and observations where biological systems have either a high mutation rate or a small population size. Even when large populations are modeled, very slightly deleterious mutations (VSDMs), can theoretically lead to continuous fitness decline.

Fisher was unquestionably one of the greatest mathematicians of the twentieth century. His fundamental theorem of natural selection was an enormous step forward, in that for the first time he linked natural selection with Mendelian genetics, which paved the way for the development of the field of population genetics. However, Fisher’s theorem was incomplete in that it did not allow for the incorporation of new mutations. In addition, Fisher’s corollary was seriously flawed in that it assumed that mutations have a net fitness effect that is essentially neutral. Our re-formulation of Fisher’s Theorem has effectively completed and corrected the theorem, such that it can now reflect biological reality.
